# Application of Transcriptomics to Enhance Early Diagnostics of Mycobacterial Infections, with an Emphasis on *Mycobacterium avium* ssp. *paratuberculosis*

**DOI:** 10.3390/vetsci6030059

**Published:** 2019-06-26

**Authors:** Marielle H. van den Esker, Ad P. Koets

**Affiliations:** 1 Department of Bacteriology and Epidemiology, Wageningen Bioveterinary Research, 8200 AB Lelystad, The Netherlands; 2 Department of Farm Animal Health, Faculty of Veterinary Medicine, Utrecht University, 3508 TD Utrecht, The Netherlands

**Keywords:** mycobacterium, host-pathogen interactions, paratuberculosis, Johne’s disease, diagnostics, biomarker, transcriptomics

## Abstract

Mycobacteria cause a wide variety of disease in human and animals. Species that infect ruminants include *M. bovis* and *M. avium* ssp. *paratuberculosis* (MAP). MAP is the causative agent of Johne’s disease in ruminants, which is a chronic granulomatous enteric infection that leads to severe economic losses worldwide. Characteristic of MAP infection is the long, latent phase in which intermittent shedding can take place, while diagnostic tests are unable to reliably detect an infection in this stage. This leads to unnoticed dissemination within herds and the presence of many undetected, silent carriers, which makes the eradication of Johne’s disease difficult. To improve the control of MAP infection, research is aimed at improving early diagnosis. Transcriptomic approaches can be applied to characterize host-pathogen interactions during infection, and to develop novel biomarkers using transcriptional profiles. Studies have focused on the identification of specific RNAs that are expressed in different infection stages, which will assist in the development and clinical implementation of early diagnostic tests.

## 1. Introduction

The genus of mycobacteria causes a wide variety of diseases in both human and animals, including tuberculosis, leprosy, and paratuberculosis. Mycobacteria are non-motile, intracellular bacteria that have a bacillary form with a distinguishing thick cell wall rich in mycolic acids [1]. Species that infect ruminants include *Mycobacterium bovis* (MB) and *Mycobacterium avium* ssp. *paratuberculosis* (MAP), of which the latter is still globally widespread and can cause Johne’s disease (JD). JD is a chronic granulomatous enteric infection that leads to lower productivity, wasting, diarrhea, and severe weight loss despite good appetite, and ultimately, to death. JD therefore results in major economic losses for stakeholders in the livestock industry, with the biggest losses in the cattle dairy industry [2]. Moreover, JD has been linked to Crohn’s disease in humans and could potentially be zoonotic, although a causal link between the isolation of MAP and Crohn’s disease has never been established [3].

MAP exposure does not always lead to infection: some animals are resilient, while at the other extreme, others become infectious, and hence proper case definition is essential [4]. Infection generally progresses in four stages depending on the severity of clinical signs, histopathological lesions and the potential for bacterial shedding [5,6]. In the first stage, animals are silently infected and display no signs of disease. Young immature stock in their first year of life are most susceptible to infection [7,8]. Diagnostic methods are unable to reliably detect infection in this phase. The second stage involves subclinical infection in which no clinical symptoms are apparent, but shedding takes place in an intermittent manner. Latent carriers remain lifelong in this phase [9], but most of the animals are still not diagnostically identified until coming close to the third stage. This third clinical phase usually only develops after several years of MAP infection in a minority of latent carriers, and clinical symptoms can be non-specific, leading to an initial misdiagnosis of JD. Direct and indirect diagnostic tests, including fecal culturing and immunological tests, are generally positive in this phase. After stage three, some animals regress into stage two and remain lifelong shedders, while the majority of symptomatic animals progress into the advanced clinical phase after weeks to several months. In this phase, health declines quickly and animals are usually culled before dying a natural death [10].

Due to the lack of proper treatment methods and efficient vaccines, current strategies are mainly based on herd-management to prevent and control transmission of infection. Transmission occurs by infected animals that increasingly shed MAP in manure and milk during the course of infection. MAP bacteria are hardy and are able to survive for extended periods in the environment, thereby infecting new animals [11,12]. Identification and removal of (sub)clinical shedders from a herd is essential to prevent further transmission. Existing diagnostic tests, like fecal culturing and antibody detection by enzyme linked immunosorbent assay (ELISA), perform well in identifying advanced stages of JD, but these tests are unable to reliably detect the majority of subclinical MAP carriers that are at risk of intermittent MAP shedding [13]. Besides, mycobacterial species, and especially *Mycobacterium avium* ssp., are closely related, which interferes with indirect diagnostic tests (e.g., antibody ELISA) [14]. For MB, the differential diagnosis of vaccinated cattle and infected animals is a challenge due to cross-reactions in indirect diagnostic tests using tuberculin or comparable crude antigens. These factors complicate the monitoring and control of MAP and MB infections, as it impedes test-and-cull strategies [15]. Research focuses on optimization of diagnosis and characterization of host properties that determine the course of infection in order to optimize management programs.

To enhance fast diagnosis and to overcome the limitations of existing diagnostic tests, research aims to identify biomarkers that can differentially detect mycobacterial infection in the subclinical, early, or pre-shedding phase. Biomarkers are defined as “any substance, structure, or process that can be measured in the body or its products and influence or predict the incidence of outcome or disease” [16]. Thus, next to being useful in diagnostics, the discovery of novel biomarkers could provide clues for the development of alternative vaccines and treatment methods of bovine and paratuberculosis. This would be an asset, as current vaccines and veterinary treatment methods are not effective [13,17].

Various strategies are used to identify possible biomarkers, including fast expanding -omics approaches. Large-scale genetic profiling by transcriptomics provides insight into regulatory mechanisms and cellular functions, as well as host responses after infection. This information may lead to the characterization of novel biomarkers or biosignatures, and thereby transcriptomics has already been demonstrated to be useful in many areas of disease [18,19]. Additionally, for MB and MAP infection, transcriptomic analysis has contributed to the discovery of putative biomarkers for early infection. Moreover, transcriptomics could expose transcriptional differences between resistant and susceptible animals. This review discusses the study of host-mycobacterial interactions by RNA expression profiling with an emphasis on MAP and the (potential) contribution of transcriptomics to biomarker discovery, and gives an overview of the possible implementations and limitations.

## 2. Pathobiology and Diagnosis of Paratuberculosis

MAP commonly enters uninfected herds when animals that appear healthy, but in reality are in the first or second stage of disease, are purchased. Moreover, wild animals, like deer, might serve as natural reservoirs, and they could introduce the disease in countries where contact between livestock and wildlife occurs [20,21,22,23]. When JD infection is subsequently detected in the clinical phase, the disease has already spread among other animals. It is estimated that for every clinical case, 5–25 subclinical shedders are present in a herd [5,24]. Diagnostic tests are unable to accurately detect these silent shedders, making an established infection within a herd difficult to eradicate. Shedding occurs in milk and manure, and MAP bacteria are transmitted mainly via ingestion by young animals.

After ingestion, MAP ends up in the ileum, where they are transcytosed through M-cells and to a lesser extent through enterocytes that are present in the Peyers Patches [25,26]. After release in the stroma, bacteria are ingested by local macrophages and dendritic cells in the lamina propria [27]. In these cells, MAP is able to resist degradation by avoiding phagosome-lysosome fusion and subsequent acidification of the phagosome. This leads to an arrest in phagosome maturation, thereby creating a niche for MAP to survive and replicate [28]. When the bacterial load becomes too high or the macrophage viability is decreasing, the host cell lyses and MAP is released in the extracellular environment. Subsequently, MAP migrates either to the intestinal lumen resulting in fecal shedding, or spreads to the draining lymph nodes or neighboring gut cells. In lymph nodes, MAP can activate T and B cells when antigens are processed and presented in major histocompatibility complex (MHC). Produced antibodies and monocytes are released in the intestine via the arterio-venous capillary bed [29]. When MAP spreads to neighboring gut cells, the infection site enlarges and granuloma can form in an attempt to shield off the infection site. In these granuloma, a balance can emerge in which MAP is confined, which keeps infected animals in a subclinical state. However, when the host cannot contain the infection (10–15% of the cases), granuloma evolve into diffuse lesions and scar tissue [30]. This causes lymph node lesions and thickening of the intestinal wall, resulting in inefficient nutrient absorption and diarrhea that deteriorates health [31].

The diagnosis of JD in live animals is based on direct detection of MAP bacteria, usually in feces, or by indirect assessment of the host response. Techniques that are most frequently used for the direct detection of MAP are molecular diagnostics, microscopy, and culturing. Culturing is regarded as the “gold standard” and it identifies the state of disease by quantifying bacterial loads in feces as these increase during the course of infection. However, MAP is fastidious and grows very slowly *in vitro*, and therefore an infection can only be excluded after months [10]. Although culturing has high specificity, the sensitivity is low in the subclinical phase due to intermittent shedding and low bacterial loads [32]. Fecal PCRs are faster than culturing, but they are sensitive to inhibitory contaminants that reduce sensitivity [33]. Recently, the accuracy of PCR tests has been improved, but sensitivities do not exceed culturing [34].

Detection of the host response is based on observation of clinical signs combined with immunological tests, including measurement of antibody production, delayed-type hypersensitivity reaction and the interferon-γ (IFN-γ) test. The application of ELISA tests is only recommended for animals in later stages of infection, since the humoral response occurs relatively late in infection. The specificity of these test is high, while the sensitivity is limited [32]. The test that most accurately recognizes MAP exposure and infection in an early stage is the IFN-γ test. This test detects the cell-mediated response after stimulation by specific antigens [35]. The antigen that is frequently used for immunological tests is Johnin purified protein derivative (PPDj), which is a filtrate of culture proteins that evokes a cell-mediated immune and antibody reaction. However, closely related species cross-react with this antigen and there is no uniformity in the production of PPDj, resulting in low sensitivity and specificity [36,37,38,39]. These cross-reactions and the occurrence of both false-positives and false-negatives limit the efficacy of current diagnostic methods. Especially, the detection of intermittent shedders in the subclinical phase is problematic. Therefore, research is aimed at finding novel biomarkers for paratuberculosis.

## 3. Novel Biomarker Discovery with Transcriptomic Technologies

A biomarker should fit certain criteria in order to be clinically implementable. It should be specific for the kind of disease, easily accessible, detect the disease rapidly and in an early stage, and be sensitive to changes in disease progression [40]. Several steps are generally taken to find novel biomarkers. First, potential candidates are identified, followed by verification and qualification in bigger patient groups. Subsequently, assays are optimized and standardized for clinical implementation and the biomarker is further validated [41].

One way to directly or indirectly identify biomarkers is with the use of transcriptomics. This technique enables the analysis of the entire set of RNA transcripts in an organism. The transcriptome consists of many different RNA species that are categorized into messenger RNAs (mRNA) and non-coding RNAs (ncRNAs). mRNA serves as template for protein synthesis by carrying genetic information from the DNA towards the ribosome. Although this was long thought to be the primary function of RNA, the prominent roles of ncRNAs are increasingly being recognized. ncRNAs consist of RNA species with a variety of functions, including gene regulation (e.g., small RNA (sRNA), microRNA (miRNA), long non-coding RNA (lncRNA)), enzyme-like activities (ribozymes, e.g., ribosomal RNA (rRNA)), and transfer of amino acids to the ribosome (transfer RNA). Their size ranges from miRNAs (21–25 bp) to long rRNA strands (thousands of bp), and they act both intra- and extracellularly [42,43,44]. The mapping of these RNAs generates valuable spatio-temporal information regarding RNA dynamics and function, and the physiological state of a cell [45].

Different techniques exist to analyze RNA expression. Before the development of large-scale transcriptomic techniques, RT-qPCR was often employed to quantify the transcription of a limited number of genes with a known sequence. RT-qPCR has a wide dynamic range and it is very robust and accurate. Additionally, the amount of starting material can be low and it is cheaper than the transcriptome approaches discussed below. In RT-qPCR, RNA is first transcribed to cDNA, which is subsequently amplified by PCR. During this PCR reaction, a fluorescently labeled probe and specialized thermal cycler are used to quantify the amount of amplified cDNA in real time. This technique is frequently employed in laboratories for molecular diagnostics in order to quantify the presence of specific DNA and RNA species. Due to its accuracy and specificity, RT-qPCR is also used to confirm results from large-scale transcriptome techniques. 

These large-scale techniques include microarrays and RNA sequencing (RNA-seq). With microarrays, known DNA probes are spotted on a chip. RNA is transcribed to fluorescently labeled cDNA, which is subsequently hybridized to the chip. The intensity of the fluorescent signal correlates to the expression level. Most common is the use of a chip containing all open reading frames (ORFs) of an organism, enabling the simultaneous measurement of thousands of gene expression levels. This technique is fast and robust, but it can only be used for known sequences and background levels can be high. RNA-seq is based on next-generation sequencing that sequences cDNA transcribed from RNA. After amplification, it is possible to construct large libraries that are sequenced and mapped onto a reference genome, creating an overview of expressed RNAs. RNA-seq has several advantages over other techniques: it sequences all of the transcripts which makes it possible to discover novel RNAs, both coding and non-coding. Moreover, it is very sensitive and accurate, and it has a high resolution down to the single nucleotide level. RNA-seq becomes increasingly affordable and since it has many benefits, it quickly replaces other methods [46].

Various types of RNA-seq can be performed. While transcriptome research traditionally focused on the characterization of either the host or pathogen transcriptome, technical advances have made it possible to perform dual transcriptome studies. By capturing and sequencing multiple transcriptomes simultaneously, the interaction of several species can be examined [47]. This gives great opportunities for the analysis of host-pathogen interactions, because RNA profiles of both organisms can be investigated. Other recent methods that enrich sRNAs have put a new perspective on the RNA landscape of many species. Deep sequencing of these samples has uncovered the presence of numerous sRNAs in all domains of life, and the functions of these sRNAs are just beginning to be elucidated [48,49]. Moreover, deep sequencing of RNA allows for very precise mapping of transcription start and termination sites, enhancing knowledge on RNA transcription and regulatory elements, such as promoter sites. Thus, RNA-seq has revolutionized our understanding of the function and diversity of RNAs.

The knowledge generated with the use of transcriptomic approaches could improve early diagnostics in various ways. Firstly, characterization of transcriptomic profiles could enhance the classification of different disease states. Exposed animals do not always progress into the shedding or the clinical phase [4], but it is largely unknown which host factors lead to MAP resilience or susceptibility and further progress of disease. Large-scale longitudinal transcriptome studies that identify differentially expressed genes in various disease stages could provide fundamental information on host characteristics that lead to altered responses and disease outcomes. This knowledge is essential to improve early diagnostics, as biomarkers that are specific for infected animals in an early stage have to be developed. Moreover, biomarkers differentiating resilient and susceptible animals could support decisions regarding culling priorities (for those at risk of progression to clinical disease and high shedding and infectiousness) or breeding preferences (favoring resilient animals).

Secondly, pathogenic virulence factors can be discovered with comparative transcriptomics. These factors are toxic to the host and allow bacteria to modify host defenses, spread within the host, and replicate intracellularly. Many virulence factors are secreted or are found on the bacterial cell surface, such as lipids and proteins, and have antigenic properties. By dissecting gene expression profiles of strains with different virulence properties, or by comparing the transcriptome of intracellular and free-living bacteria, induced virulence genes and pathways can be pinpointed. Hence, comparative gene expression profiling could identify potential antigens for further validation as more specific biomarkers.

A third approach characterizes unique RNA biosignatures, which comprise a collection of host RNAs that are specifically expressed during infection with a certain type of pathogen. These biosignatures can be based on differentially expressed genes (DEGs) derived from circulating blood cells, but also on ncRNAs that are present in blood cells or extracellular vesicles (ECVs). ECVs contain secreted RNAs (seRNAs) that play an important role in cell-to-cell communication, and their expression and secretion depends on the physiological state of the cell. Sequencing of these RNAs can therefore provide very specific information on the cell’s situation, making them promising diagnostic targets. Several seRNAs can be isolated from extracellular fluids, like serum, milk, urine and saliva, and the stability of these circulating RNAs is high, even when stored under suboptimal conditions [50,51,52]. Extraction of some of these extracellular fluids is non-invasive, which is a big advantage in clinical implementation.

Recently, it has become clear that bacteria also secrete vesicles that contribute to extracellular inter- and intrakingdom communication. These vesicles contain several components, such as DNA, lipoproteins, signaling molecules and RNAs [53]. RNAs in ECVs are often enriched in small, ncRNAs with stable secondary structures that potentially regulate gene expression in target cells directly and indirectly by influencing the transcription, translation, processing, or stability of mRNAs [54]. The isolation of these secreted seRNAs from body fluids of infected hosts would directly demonstrate bacterial presence, and could thus be a promising target for biomarker development [55]. Follow-up studies should investigate the stability, function and omnipresence of these bacterial seRNAs in order to determine whether they are suitable biosignature candidates.

After biomarker candidates are identified by transcriptomics and subsequently validated in a clinical setting, a novel diagnostic test can be developed. The design of such a test depends on the type of marker. The presence of antigens encoded by identified virulence factors could be demonstrated by serological tests. Circulating RNA can be detected and quantified by RT-qPCR or microarrays. Sometimes, a single RNA might be enough to detect disease, but selection of multiple conditionally dependent expressed RNAs could improve the specificity of such a test, and thereby improve the diagnostic potential. Table 1 gives a summary of the possible detected markers by transcriptomics.

## 4. Mycobacterial RNAs Induced during Infection Could Lead to Novel Antigen Identification

Mycobacterial invasion depends on silent host cell invasion, and many classic bacterial virulence factors are lacking in this genus in order to avoid or delay host cell recognition. Transcriptome studies therefore have a prominent role in the identification of unknown factors that are required for virulence and successful host colonization. MAP pathogenicity depends on successful entry and survival within the host cell by establishing an intracellular replicative niche. Therefore, phagosome-lysosome fusion and the toxic effects of compounds produced during phagocytosis, such as reactive oxygen species, have to be avoided. Many factors that play a role in mycobacterial pathogenesis have been identified and have been reviewed elsewhere [65].

For MAP infection specifically, several *in vitro* transcriptome studies have been performed that led to insight in pathology and intracellular survival pathways. These studies have mainly been performed by infecting macrophages with MAP and comparing gene expression from intracellular MAP to MAP grown in liquid cultures [66,67]. DEGs were involved in cell wall biosynthesis, combatting oxidative stress, and adaptation to an anoxic environment and nutrient starvation. Moreover, a dual RNA-seq study revealed that MAP adapts mycolic acid metabolism and DNA repair to enhance intracellular survival [68]. Bacteria isolated from intestinal lesions in subclinical affected cows also showed adapted metabolism, cell envelope biogenesis, and increased latency gene expression [69]. Mutational studies have indicated that two sigma factors, SigH and SigL, are mandatory for successful host colonization, as the deletion of these transcription factors severely attenuated MAP strains [70,71]. Gene expression analysis of *sigH* and *sigL* deletion mutant strains revealed that the transcription of genes involved in oxidative stress and virulence were affected. These transcriptome studies could lead to the characterization of novel virulence factors, followed by more focused, hypothesis-driven research that identifies novel biomarkers. Additionally, attenuated strains are potential novel vaccine candidates against JD.

For MAP, complementary research is required to validate which genes serve as antigens, but for MB several new antigens have been characterized by a transcriptome approach. The diagnosis of MB infection is complicated by cross-reactions with vaccinated cattle. Therefore, antigens that are able to differentiate between vaccinated and infected cows are demanded. Genes transcribed in MB, but not in bacille Calmette-Guérin (BCG; the only available tuberculosis vaccine strain), were listed to select potential differentiating antigens. MPB70 and MPB83 were constitutively expressed to high levels in MB, but only very lowly in some BCG strains [57]. Therefore, these proteins are among the most frequently investigated antigens for the differential diagnosis of bovine tuberculosis and they have been examined as diagnostic biomarkers [72,73,74]. Another study also screened for highly expressed mycobacterial genes that are lowly expressed in the BCG strain, and identified Rv3615c as a useful differential diagnostic antigen [56]. This antigen has also been further investigated for its clinical potential to distinguish between MB, BCG vaccinated and uninfected cows [75,76,77]. Large scale validation of these antigens is ongoing in order to determine their specificity and sensitivity.

With the development of RNA-seq, attention is increasingly being shifted towards ncRNA species, such as bacterial sRNAs. sRNAs are 50–250 bp long and they post-transcriptionally regulate mRNA expression by binding to complementary seeding regions of 6–8 bp in the mRNA strand [48]. sRNAs evolve rapidly, and many sRNAs are species- or even strain-specific [78]. Intracellular bacteria located inside eukaryotic cells produce defined sRNA species, but the contribution of these sRNAs in infection is yet unclear [79]. Pathogenic seRNAs in ECVs are other fascinating research targets, but until now, investigations mainly focused on the role of seRNA in viral infections. On the bacterial side, seRNA expression during *Mycobacterium tuberculosis* infection has most often been investigated. Mycobacterial sRNAs have been detected in blood plasma of patients with active tuberculosis, giving prospects for the use of secreted sRNA as biomarker in tuberculosis and potentially also in other infectious bacterial diseases, like JD [80].

sRNA mapping and function annotation facilitates RNA-seq data analysis. Several studies have concentrated on mapping the sRNA landscape of *M. tuberculosis* and other mycobacterial species, but the non-coding transcriptome of MAP has until now not been explored [81,82,83,84,85]. In MB, 34 novel sRNAs were detected that were also conserved in closely related *M. tuberculosis* and *M. smegmatis* strains [86]. Although these strains are phylogenetically more distant to MAP, these could be conserved throughout the genus. Deep sequencing of the highly related *Mycobacterium avium* ssp *avium* identified 97 ncRNAs and the high genetic homology between *M. avium* species makes it plausible that these ncRNAs are conserved in MAP as well [87]. Next to sRNA mapping by RNA-seq, *in silico* predictions of mycobacterial sRNAs have been made [88,89]. Bioinformatics can also be used to predict the mRNA binding partners of sRNAs. These predictions give valuable information, but should be experimentally validated, as false-positive and negatives are known to exist [90]. Therefore, the mapping of sRNAs by large-scale deep sequencing is indispensable, especially since the discovery of MAP-specific sRNAs could enhance diagnostics. Moreover, the functions of most sRNAs remain elusive, and future studies should focus on unraveling the biological role of the identified sRNAs.

## 5. The Host Response during Mycobacterial Infections

### 5.1. RNA Expression Analysis of Infected Host Cells Elucidates Virulence Pathways and Host Cell Responses

MAP infection triggers a response that modifies gene expression of host cells. Intestinal macrophages are the preferred initial colonization site of MAP, and macrophage cell lines are often employed as a model system to mimic infection conditions *in vitro*. Generally, these transcriptome studies have revealed that MAP invasion alters the expression of genes that are involved in vacuole maintenance, apoptotic pathways and the immune response of the host cell [68,91,92,93,94] depending on the time after infection. Early in infection (2 h post infection), pro-inflammatory mediators like interferons were activated, but after prolonged incubation, interferons and the JAK-STAT pathway were repressed [92,93]. Moreover, several anti-inflammatory mediators, including the interleukin-10 (IL-10) signaling pathways, were induced, which is characteristic for MAP infections and has been confirmed by transcriptome studies that analyzed gene expression in peripheral blood mononuclear cells (PBMC) [93,94,95,96]. Thus, by modulating the immune response, MAP promotes bacterial survival and establishes an intracellular niche.

Other studies analyzed RNA expression in ileal tissues extracted from infected animals and compared it to genes expressed in uninfected tissues to characterize the host reaction on MAP infection. A study of early stage infection in experimentally infected newborn calves revealed that MAP infection may lead to over-proliferation of endothelial cells one month post-infection [97]. Moreover, nine miRNAs that might be involved in host responses to MAP infection, including bacterial recognition and regulation of the inflammatory response, were differentially expressed. In the ileocecal valve of naturally infected cows, many immunological pathways were altered in clinical diseased versus uninfected cows, but differences between subclinical and uninfected cows were more subtle and mainly involved metabolic genes [98]. These results using tissues from naturally infected animals also emphasize the need to carefully define and describe case definitions to allow for comparisons between studies. Inoculation of ligated jejuno-ileal loops in neonatal calves with MAP and its closely related, but less pathogenic sibling *Mycobacterium avium* ssp. *avium* (MAA) showed that MAP manipulates epithelial cell proliferation and weakens the mucosal membrane to promote access to the ileum [99]. Moreover, calcium signaling was inhibited during MAP infection, which corresponds to decreased phagosome-lysosome fusion [100]. Finally, the immune response was biased towards the Th2 cell immune response during MAP incubation, which could lead to persistent infection, while MAA infection led to a strong bias towards the Th1 immune response, resulting in a transient infection. Such comparative studies are essential for the detection of pathogen-specific based gene profiles.

Although most research has been performed in dairy cattle, some studies focused on the genetic host response of small ruminants after MAP infection. In comparison to cows, the disease progresses more insidiously in small ruminants like sheep, and clinical symptoms such as watery diarrhea are often lacking. In sheep, two disease states can be distinguished: a paucibacillary (P) and multibacillary (M) form. P pathogenesis is characterized by lesions consisting of lymphocytes and relatively few bacteria, while the M form comprises heavily infected macrophages, leading to high shedding and declining health. The development of one of both forms has been linked to differences in the host immune response, including increased Th1 and IFNγ production in the P form and induced Th2 activation in the M form [101]. Several transcriptome studies have focused on the characterization of the host response during these different infection states. Chemokines and cytokines were differentially expressed in terminal ileum sections during both disease states, with TLR-2 being greatly induced in M lesions, which could induce IL-10 transcription [102]. Transcriptome analysis of the ileocaecal lymph node, the major immune-inductive site for the terminal ileum, suggested that the development of M pathology depends on a cumulative decline into immune dysfunction, as no fundamental differences in gene expression patterns between the M and P disease were found, and no shift in T cell genes was apparent [103,104]. These results support the hypothesis that a general exhaustion of the immune system causes health deterioration.

Besides obtaining information on host gene transcription during infection, genetic profiling can provide insight into the transcriptomic background, leading to varying host characteristics. For instance, it is unknown why some MAP infected ruminants progress into the clinical phase, while other related members are resilient or remain silently infected and never progress into further stages. In red deer, this has been explored by global gene expression profiling of macrophages derived from susceptible and resistant animals. These were infected and compared to uninfected macrophages [105]. Susceptible animals had substantially more DEGs when compared to resistant animals, and many genes, including chemokines and genes related to chemotaxis, were overexpressed in susceptible animals. Macrophages isolated from cattle that were naturally susceptible to MB infection displayed an altered transcriptome profile when compared to resistant cattle as well [106,107]. Especially pro-inflammatory pathways were stronger induced in resistant animals, resulting in improved intracellular control to MB infection. These studies that focus on the different transcriptional behaviors of resistant and susceptible animals could aid in correct classification of disease stages and the development of stage-specific biomarkers.

### 5.2. Eukaryotic Circulating RNAs Are Promising Novel Biomarkers

Known protein-coding gene exons make up less than 3% of the human genome, and for a long time it was a mystery what the function of the remaining 97% was. Often, it was seen as junk DNA. With the use of deep sequencing, it has become clear that around 90% of the human genome is transcribed. It is now becoming evident that many previously unrecognized ncRNAs are transcribed, although some scientist believe that a big percentage represents transcriptional noise [108,109]. The functions and mechanisms of these ncRNAs are just beginning to be characterized, and the influence of ncRNAs on all cellular levels becomes increasingly evident. Many fulfill a function on all levels of gene regulation, including the control of chromosome dynamics, RNA editing, and mRNA destruction [110]. Growing evidence suggests that ncRNAs are abundant in other eukaryotes as well and play similar roles as in human. Hence, RNA-seq has opened up an entire level of new transcriptomic research.

Promising biomarkers include stable circulating RNAs that can be isolated in a non-invasive manner from body fluids, such as miRNAs. miRNAs are transcribed from the genome as pri-miRNA, followed by cleavage to pre-miRNA. Pre-miRNA is exported from the nucleus and further processed to a 21–25 bp strand of miRNA. miRNAs mediate mRNA silencing, e.g., by silencing transcription or by inducing mRNA degradation, and thereby play an important role in gene regulation. It has been estimated that approximately 60% of genes are regulated by miRNAs in human. Since seeding regions are short, one miRNA can have multiple targets [111]. miRNA transcription depends on the physiological status of the cell, and the qualification and quantification of miRNAs can therefore provide information on its immunological status. In the last years, much research has focused on identifying disease-specific miRNA biosignatures to facilitate diagnosis and prognosis, and to monitor therapy efficacy.

Several studies have characterized miRNA expression profiles during MAP infection derived from blood from infected animals. In an attempt to identify early biomarkers, Farrell et al. experimentally challenged three to six week old calves with MAP, and extracted serum half a year post-infection in order to analyze miRNA profiles. However, no miRNAs were significantly differentially expressed in MAP infected animals compared to uninfected controls, and only the abundance of age-related miRNAs was altered in the pre- and post-infection phase [112]. These results entirely correspond to another study that analyzed miRNA expression in calves [52]. Gupta and colleagues isolated serum from older (three to five year old) uninfected and naturally MAP infected cows and performed a human-based miRNA measurement [113]. This approach exposed 26 differentially expressed miRNAs, of which 13 miRNAs had not yet been characterized in cattle. Four distinct clusters of miRNA were discerned based on the severity of the disease. Four of these miRNAs may have the potential to discriminate moderate and severely infected animals from uninfected animals, which could indicate a starting point for further biomarker development.

Malvisi et al. used whole blood to investigate miRNA levels of cows in different stages of MAP infection, consisting of an infected (ELISA and culture positive), exposed (but ELISA negative) and uninfected group [114]. These cows were older (four to five years old) and naturally infected. Nine miRNAs were identified that were differentially expressed between the infected and uninfected group and eight between the exposed and uninfected group. These miRNAs were mainly involved in regulating immune responses, which suggests that miRNAs have a regulatory role in the host response after MAP exposure. This was also confirmed by another study that employed a mouse model to demonstrate that miRNA-27a-3p was downregulated during MAP infection *in vivo* and *in vitro* [115]. This miRNA inhibits IL-10 expression, and thereby regulates innate immune responses during MAP infection. Besides expanding knowledge of the regulatory functions of miRNAs, these markers could be further developed into diagnostic tests.

Next to the characterization of circulating miRNAs, differentially expressed mRNAs in whole blood could provide suitable targets for early diagnostic tests. Although being less stable than circulating miRNA, mRNA can quickly be extracted by experienced laboratory staff and easily quantified by RT-qPCR. Many studies have focused on differential gene expression in whole blood, PBMCs, or white blood cells with the aim of increasing immunological knowledge, but also to trace potential biomarkers [116,117,118,119,120,121]. Over a decade ago, microarrays already identified a unique gene expression profile in PBMCs and total leukocytes that were able to distinguish MAP infected from uninfected cattle [122,123]. However, gene expression appears to be very condition dependent, and the list of identified DEGs is often exhausting. To extract suitable candidate marker genes that are uniquely expressed during a specific infection phase requires extensive follow-up research performed under different conditions. One attempt to identify a subset of marker genes first traced DEGs *in vitro* in murine macrophages, and in an *in vivo* mice model [124,125]. These results were further extrapolated to *in vivo* bovine models, resulting in a subset of eight marker genes (*Timp1*, *Hp, Serpine1*, *Tfrc*, *Mmp9*, *Defb1*, *Defb10*, *and S100a8*) in whole blood that could be used for the diagnosis of JD, including early subclinical stages [126,127]. Interestingly, a recent study suggested that ovine genetic profiles isolated from white blood cells can predict disease outcome after MAP exposure, and resilient and diseased sheep could be differentiated based on these profiles [128]. These differentially regulated genes could represent a biosignature that is specific for MAP exposure, or of the specified disease or resilience outcome.

mRNAs and miRNAs have until now most frequently been investigated for their use in diagnostics, but recently discovered lncRNAs are also potential biomarkers. LncRNAs are longer than 200 basepairs, and regulate mRNA expression at the transcriptional, post-transcriptional and epigenetic level. Their numbers have been estimated to equal mRNAs, though the precise functions of lncRNAs have so far rarely been characterized. Similar to miRNA, lncRNA can be secreted, but their expression seems to be more time- and tissue specific. Therefore, attention has shifted towards the use of these RNAs as biomarkers. For MAP, one study has focused on the contribution of lncRNAs during infection [94]. 397 lncRNAs were identified, of which 38 were differentially expressed during infection. These lncRNAs were mainly involved in the regulation of immunological pathways. This study is the first step in deepening the knowledge regarding lncRNAs in MAP infection, but research in this field is still in its infancy. The regulatory functions of lncRNAs during bacterial infection and their application as biomarker remain to be further established. Moreover, although eukaryotic seRNAs are very stable and appear to be promising diagnostic targets, disease-specific lncRNAs or miRNAs probably do not exist. Most of these RNAs have general regulatory roles and are widely expressed in an unspecific manner, and lnc/miRNA-based tests must therefore measure a wide panel of differentially expressed RNA species.

## 6. Practical Considerations for the Design of Transcriptome Experiments

Transcriptome experiments require a proper set-up and precise preparation. The first step involves the design of experimental conditions, including the selection of media and/or model system. When the goal is to approach reality as closely as possible, it is important to choose a mimicking condition accordingly. Many *in vitro* studies use murine macrophage cells to investigate intracellular infections. These cell lines are accessible and easy to maintain, and the step to *in vivo* models (i.e., experimental animals) is smaller when using mice models compared to e.g., cattle. However, it can be questioned how representative these models are, as macrophages from different species can vary significantly in cellular characteristics [129,130]. Moreover, immortal cell lines can harbor different properties compared to macrophages derived from primary cells [131,132]. This should be considered when choosing a model system and before extrapolating results to *in vivo* macrophage function.

Although macrophage infection assays could provide initial suggestions for interesting biomarkers, the real environment is much more complex. This often leads to disappointing results when the results are further evaluated *in vivo*. To limit the use of experimental animals and obtain more predictive results, other *in vitro* models that approach reality more closely are increasingly being developed. Such models include organoids that mimic the three-dimensional structure of e.g., the lung and gut *in vitro* [133,134]. Moreover, *in vitro* models of granuloma have been developed by infecting PBMCs in a matrix with mycobacteria. These models were shown to be representative for the cellular composition, cytokine production, and bacterial responses of *in vivo* granuloma [135]. Therefore, these granuloma could be used to more reliably identify biomarkers *in vitro* and thereby reduce the use of experimental animals.

Most host-pathogen transcriptome studies use a big amount of infected cells in order to obtain enough RNA. This creates a comprehensive overview of the average gene expression in a population, but it is not always a proper representation of individual cell expression, as heterogeneity in RNA transcription and time of infection can occur [136]. For example, Helaine et al. showed that during infection with the facultative intracellular bacterium *Salmonella*, the bacterial population can be divided in two pheno- and genotypically distinct fractions. Part of the population consists of non-replicative persister cells that exhibit completely different behavior than their non-dormant siblings [137]. Increased accessibility of single cell RNA-sequencing techniques could in the future aid in differentiating additional pathogenic subpopulations that are probably more abundant than currently acknowledged.

When the goal of a transcriptome experiment is to identify unique, specific biomarkers, the use of a proper control group is essential. Often, infected and uninfected cells—or infected and uninfected animals—are compared to evaluate differentially expressed RNAs. These studies offer useful insight in host-pathogen interactions, but sometimes biosignatures are deduced from differentially expressed RNAs as well. Although these experiments provide an initial profile for further analysis, infection could also evoke a more universal response in host cells, which is not distinctive between two related pathogens. Therefore, gene expression of infected cells should ultimately be compared to cells infected with other resembling pathogens in order to be able to discriminate between other diseases. For MAP, comparisons with other mycobacterial infections could establish the specificity of such a signature.

High quality RNA and libraries should be prepared after sampling is accomplished. Many different commercial kits are available for RNA isolation, and the RNA species of interest determine which are the most suitable. Trizol is often used to extract total RNA of both pro- and eukaryotes, but short RNA structures with low GC content are selectively lost with this procedure [138]. Therefore, Trizol protocols should be adapted for the isolation of sRNAs and miRNAs. When preparing a library, choices should be made regarding stranded or non-stranded sequencing, and single versus paired-end sequencing. The goal of the experiment defines which methods are preferred, but a quality control should be performed after each step to ensure high RNA quality.

A final note on the design of an optimal transcriptome experiment includes the costs that are associated with RNA sequencing. The amount of biological replicates and reading depth required to sequence the sample determine a big part of the expense. Studies have indicated that, if a choice has to be made, a higher number of replicates is preferred over an increase of the sequencing depth, especially when one wants to detect DEGs [139,140,141]. A small sample size could lead to low reproducibility due to the relatively large contribution of accidental outliers. One solution is to pool samples in order to reduce costs, although this could increase the technical error rate. The optimal amount of biological replicates depends on the experiment and should be individually determined, but the minimum is estimated to be at least three to six [140,142]. To save costs, pilot experiments can be performed that are extended after a successful trial.

After the sequencing process, software analysis is performed to evaluate the obtained data. Several challenges can occur in this stage, and expertise in bioinformatics and statistics is required for proper data handling. Data analysis basically follows one general outline: after quality assessment, sequences are trimmed and mapped onto a reference genome. Subsequently, the data is normalized and with further downstream processing, RNA counts, DEGs, and altered cellular pathways can be extracted. At the moment, many different pipelines and scripts are publically available. Most of these scripts are written in R, and DESeq and edgeR are most commonly used for DEG analysis [143,144]. Besides varying approaches in pipeline analysis, there is no consensus regarding the storage and availability of data. Since the use of RNA-seq is rapidly increasing, the implementation of cloud computing and storage for such big data is indispensable.

## 7. Future Outlook

The use of transcriptomics in the discovery of novel biomarkers is quickly expanding, and some RNA-based tests have already been implemented in clinical practice. The step from laboratory detection of RNA signatures towards clinical practice involves thorough testing and upscaling. Initial RNA expression profiles are often mainly derived from *in vitro* model systems. When a putative biosignature is found, it is tested whether similar RNAs are expressed *in vivo*. In this stage, the accuracy and reliability of a potential test is evaluated. If positive, clinical validity should be assessed, and sensitivity and specificity can be verified in bigger patient groups. This is a critical milestone that eliminates many candidates, as the profiles identified in preliminary studies can be unspecific and dependent on the platform used [145]. Practical considerations ultimately influence the clinical utility, including uniqueness of the test, economic feasibility and quality assurance [146].

Until now, most progress in RNA biomarker discovery has been made in oncology, where several (mi)RNA biosignatures are clinically used to distinguish between tumor types, identify the stage of cancer, and to monitor the therapeutic efficacy [147]. For example, the expression of 70 genes in breast tumors is analyzed in a prognostic test, called MammaPrint, to help predict the risk of recurrence [148]. Other prognostic, predictive, and diagnostic tests are available for e.g., prostate, colon and thyroid cancer [146]. For the diagnosis of human genetic disease, microarrays havebecome indispensable as well. In veterinary clinical settings, RT-qPCR is widely implemented, while the application of microarrays is so far limited in routine diagnostics [149].

Advances in RNA-seq technologies could aid in the development of novel, more accurate biomarkers. With the use of single-cell RNA-seq, very specific profiles can be acquired from cells during different stages of infection. For example, a fluorescent marker can be used to isolate cells with different bacterial loads or in a different developmental stage (e.g., acute vs. chronic) and subsequently, RNA can be isolated and sequenced [150]. This will provide useful information that discerns between cells within a population and has for instance led to the identification of HIV permissive conditions of host cells and biomarkers that determine CD4^+^ cell permissiveness [151]. Single RNA-seq of resistant versus sensitive host cells to MAP infection could likewise lead to the detection of features that make host cells sensitive to bacterial colonization. Moreover, characterization of bacterial genes expressed during different stages of infection could identify biomarkers for acute and more advanced stages of infection.

One of the biggest obstacles in sequencing small amounts of RNA is the need for cDNA conversion and subsequent PCR amplification, which could induce a bias. These biases become bigger when RNA from a single cell is sequenced, as very high amplification rates are needed [152]. A new technique enabling direct sequencing of RNA circumvents these steps by directly sequencing the isolated RNA strands [153]. It utilizes a nanopore platform that is strand-specific and is compatible with very long read lengths. Direct sequencing is a great step forward, but caution should be taken with regards to the stability of RNA. RNA strands are prone to degradation and often have a short lifetime, depending on the RNA species. Additional analytic experiments should show whether all RNA strands are sequenced or whether (some specific) RNA species are lost in the sequencing process, leading to a bias in gene counts.

At the moment, RNA-seq is mostly employed for the discovery of novel biomarker candidates, which are further translated in established diagnostic assays, like microarrays or RT-qPCR. However, with the upsurge of NGS technologies, the future implementation of RNA-seq in clinical situations becomes more realistic. The accuracy, robustness, and sensitivity of this technique, together with its open nature not limited to certain transcripts or organisms, provides great clinical benefits. A few challenges remain regarding clinical implementation, as the interpretation of RNA-seq results stemming from the discovery of e.g., unannotated transcripts or single nucleotide polymorphisms (SNPs) could raise uncertainties. Besides, the costs of RNA-seq form until now a limiting factor. Proper schooling and sample handling is crucial, and clinicians are often incompletely educated in bioinformatics and statistics. Therefore, an easily applicable pipeline should be delivered in order to avoid ambiguity. Standardization of RNA-seq protocols and tests in clinically relevant conditions is imperative.

Additionally, novel diagnostic platforms are being developed that are suitable for rapid point-of-care detection of low abundance RNA-based biomarkers. Major factors that complicate the clinical adoption of (mi)RNA based assays are the extensive processing steps required upstream of RNA quantification, such as RNA purification from ECVs and amplification. These steps are expensive, time intensive and require proper training of laboratory staff, because samples are prone to degradation and quantities are often low. Digital PCR is a technique with improved precision that eliminates the need for standard curves and normalization strategies, and could therefore be useful for the detection of low RNA concentrations [154]. However, upstream steps are still necessary. Therefore, the development of new assays aimed at the quick and affordable detection of RNAs at ultralow concentrations that omit upstream sample processing steps are a big advancement. Recent innovations include purification and amplification-free technologies, thereby excluding traditional cDNA generation by using the miRNA itself as a primer [155]. These one-step RNA detection methods provide promising future point-of-care assays.

An example includes the application of microfluidic devices that detect RNAs using electrochemical or fluorescent detection systems without the need of prior PCR amplification. For the early diagnosis of epilepsy, a one-step RNA detector employing a microfluidic disc was developed that allowed for fast and simple quantification of a miRNA biomarker in unprocessed biofluids, including plasma [156]. This technique is based on the use of probe-functionalized platinum nanoparticles that electrochemically detect the miRNA target strand, without the requirement of prior amplification. Another system based on microfluidic cards and isothermal base-stacking amplification allowed for direct one-step miRNA detection from body fluids in <60 min [157]. The results of a fluorescent-based microfluidic device that was tested to diagnose early breast-cancer stages by screening miRNA-21 quantities concurred with RT-qPCR, while working faster and more straightforward [158]. Another electrochemical biosensor with screen printed electrodes detected miRNA in attomolar concentrations in human serum [159]. Preliminary results indicate that such devices offer quick, reliable, and straightforward tests that are suitable for point-of-care applications in mass-screening programs, which suggests that they might also be suitable for rapid on-farm detection of MAP.

The fast developments in RNA-sequencing promise a great future for both the fundamental discovery of novel biomarkers by RNA-seq, as well as clinical implementation of RNA-based tests. Although work is required on standardization and validation of working methods and pipelines, much progress has been made in the development of biosignatures and testing platforms. Particularly, host RNA profiles are already used in diagnostic, prognostic and predictive tests in human oncology, but pathogen derived seRNAs are also potential targets. Moreover, the development of novel point-of-care assays will facilitate clinical implementation. The increased accessibility and affordability of deep sequencing techniques promises a transfer to veterinary settings as well.

## 8. Conclusions

Diagnostic tests for mycobacterial infections and especially MAP infection are only reliable in the late subclinical and clinical phase of disease, while diagnosing early shedders remains challenging. Therefore, more specific tests are demanded that diagnose infection in an early stage, and research is focused on the identification of novel biomarkers. Transcriptomic techniques could aid in this quest by obtaining infection-specific RNA expression profiles of the pathogen and/or host cell. Bacterial transcriptome analysis could lead to the identification of novel antigens, but circulating RNA species are promising targets as well. These RNAs are stably secreted in extracellular body fluids and can thus be isolated in a non-invasive way. Circulating RNAs can originate from the pathogen or the host, and include species-specific bacterial sRNAs and differentially expressed host RNAs, such as miRNA and lncRNA. These host ncRNAs could depict a pathogen-specific host response and give a good representation of the infection status. RNA-based tests are already clinically implemented in oncology, and related research is ongoing in other fields, including intracellular diseases, like mycobacterial infections. Future research should focus on validating the specificity and sensitivity of RNA-based tests, and on practical considerations, such as workflow standardization and the development of improved testing platforms.

## Figures and Tables

**Table 1:** Possible diagnostic biomarkers detected by transcriptomics.

Biomarker	Description	References
Antigens	Novel bacterial virulence factors can be identified by analysis of differentially expressed bacterial genes under infectious conditions. These antigens could evoke an immune response in the host that can be detected by immunological assays	[56,57]
Circulating, secreted host RNA (miRNA, lncRNA)	Extracellular RNA secreted in body fluids is easily accessible and very stable. Disease specific RNA signatures can be developed into diagnostic arrays, RT-qPCR tests or novel point-of-care tests	[58,59]
Blood cell-derived RNA	Infection may induce a specific immune-driven RNA expression profile in blood cells. These profiles can be traced by transcriptomics and developed into a biosignature-based test	[60,61,62,63]
Bacterial secreted RNA	Bacteria secrete RNA in extracellular vesicles that circulate in the host. When these RNAs can consistently be detected, diagnostic assays could be developed that directly recognize an infection	[54,64]
